# Mitochondria-associated endoplasmic reticulum membranes (MAMs) and their role in glaucomatous retinal ganglion cell degeneration—a mini review

**DOI:** 10.3389/fnins.2023.1198343

**Published:** 2023-05-12

**Authors:** Jennifer H. Pham, Dorota L. Stankowska

**Affiliations:** ^1^Department of Pharmacology and Neuroscience, University of North Texas Health Science Center, Fort Worth, TX, United States; ^2^North Texas Eye Research Institute, University of North Texas Health Science Center, Fort Worth, TX, United States

**Keywords:** mitochondria, endoplasmic reticulum, MAMs, retinal ganglion cells, glaucoma, oxidative stress, ER stress, inflammation

## Abstract

Glaucoma is a leading cause of blindness worldwide, commonly associated with elevated intraocular pressure (IOP), leading to degeneration of the optic nerve and death of retinal ganglion cells, the output neurons in the eye. In recent years, many studies have implicated mitochondrial dysfunction as a crucial player in glaucomatous neurodegeneration. Mitochondrial function has been an increasingly researched topic in glaucoma, given its vital role in bioenergetics and propagation of action potentials. One of the most metabolically active tissues in the body characterized by high oxygen consumption is the retina, particularly the retinal ganglion cells (RGCs). RGCs, which have long axons that extend from the eyes to the brain, rely heavily on the energy generated by oxidative phosphorylation for signal transduction, rendering them more vulnerable to oxidative damage. In various glaucoma models, mitochondrial dysfunction and stress from protein aggregates in the endoplasmic reticulum (ER) have been observed in the RGCs. However, it has been shown that the two organelles are connected through a network called mitochondria-associated ER membranes (MAMs); hence this crosstalk in a pathophysiological condition such as glaucoma should be evaluated. Here, we review the current literature suggestive of mitochondrial and ER stress related to glaucoma, indicating potential cross-signaling and the potential roles of MAMs.

## 1. Introduction

As the second leading cause of blindness worldwide, there are an estimated 80 million patients, and glaucoma is predicted to affect 112 million people by 2040 (Tham et al., 2014; Allison et al., 2020). Characterized by retinal ganglion cell (RGC) degeneration and optic neuropathy, glaucoma causes gradual loss of peripheral vision, which delays diagnosis since over 50% of people affected are unaware that they have the condition (Topouzis et al., 2008; Gupta et al., 2022). Current therapies focus on reducing elevated intraocular pressure (IOP), a significant risk factor for the most common form of the disease: primary open-angle glaucoma (POAG) (Martinez and Peplow, 2022). While these treatments can effectively reduce IOP, progressive loss of RGCs still occurs (Martinez and Peplow, 2022). Consequently, it is crucial to conduct additional research and gain a deeper understanding of the diverse underlying factors that result in the death of RGCs in glaucoma. This will aid in the development of novel neuroprotective strategies for treating glaucoma.

Retinal ganglion cells integrate and transmit visual signals from the eyes to the brain in the central nervous system. With long axons unmyelinated in the prelaminar region before they exit the eyes, RGCs have a high energy demand to transmit these signals, which is met by high oxygen consumption and oxidative phosphorylation. This high demand makes them more vulnerable to oxidative stress from reactive oxygen species (ROS) generated during ATP production (Kang et al., 2021). In glaucoma, like other age-related diseases, mitochondrial function and the availability of antioxidants are reduced, producing a higher amount of ROS (Garcia-Medina et al., 2020). The imbalance between levels of antioxidants and ROS induces damage to the mitochondria, which abounds in RGCs that rely on oxidative metabolism.

The endoplasmic reticulum (ER) has many functions within a cell, including storing calcium ions (Ca^2+^) and responding to unfolded proteins through the unfolded protein response (UPR^ER^) pathway (Treiman, 2002; Schwarz and Blower, 2016; Hurley et al., 2022). In pathological conditions, the aggregation of these unfolded or misfolded proteins leads to ER stress (Lin et al., 2008). The presence of ER stress has been detected in glaucoma in various areas of the eyes, including the trabecular meshwork, the retina, and RGCs, specifically (Hurley et al., 2022).

The mitochondria and ER work together for various biochemical processes in the cells, with Ca^2+^ playing a significant role as a signaling molecule for essential pathways such as autophagy and apoptosis (Hurley et al., 2022). These organelles accomplish these tasks through the help of contact sites (anchored by mitochondrial and ER proteins where they can communicate), known as mitochondria-associated ER membranes (MAMs)/mitochondria-endoplasmic reticulum contact sites (MERCs). With both mitochondrial and ER dysfunction being observed in glaucomatous optic neuropathy, it is of interest to understand the possible role(s) that MAMs play in this age-related neurodegenerative condition as it has been shown to play in others. In this review, we evaluate the research on cellular stress responses involving the mitochondria and ER in glaucomatous RGC degeneration to see the crosstalk between the two organelles and any potential involvement of MAMs.

## 2. Cellular stress responses in glaucomatous retinal ganglion cell degeneration

In a multifactorial condition like glaucoma, the degeneration of RGCs can be triggered by various forms of stress, including oxidative stress, ER stress, inflammation, and metabolic stress. Although their involvement in RGC degeneration is not fully understood, several mechanisms have been proposed based on experimental data.

### 2.1. Mitochondrial dysfunction

Reactive oxygen species are mainly produced by electron leaks in the mitochondrial electron transport chain, resulting in a partial reduction of molecular oxygen molecules. Another source is ER stress, which contributes to about 25% of ROS production through processes such as oxidative protein folding involving the ER oxidoreductin 1 (Ero1) protein (Tu and Weissman, 2004; Zhao et al., 2019; Hurley et al., 2022). When endogenous antioxidants (superoxide dismutase, catalase, etc.) are depleted in glaucoma, ROS cannot be cleared, leading to oxidative damage to DNA, proteins, and other cellular components. This damage can further lead to mitochondrial dysfunction, decreased mitophagy, and cell death (Guo et al., 2013; Hurley et al., 2022; Pham et al., 2022). However, it is important to note that at physiological levels, ROS can also function as a secondary messenger to modulate protein functions through oxidative post-translational modifications. This process has been observed in the retina of rat eyes with IOP elevation (Tezel et al., 2005; Wall et al., 2012; Hurley et al., 2022).

Oxidative stress can be induced by various factors, including inflammation, ischemia, and axonal transport deficits that deprive RGCs of essential nutrients. Like the ER, the mitochondria also have their UPR (UPR^mt^) to respond to perturbations to the mitochondrial protein import, including excessive ROS and accumulation of misfolded proteins (Shpilka and Haynes, 2018). In the case of the mitochondria, activating transcription factor associated with stress (ATFS-1) will translocate to the nucleus and activate UPR^mt^ (Shpilka and Haynes, 2018).

There are three other basic leucine zipper (bZIP) transcription factors associated with mitochondrial dysfunction response: C/EBP homologous protein (CHOP, also known as DDIT3), activating transcription factors 4 and 5 (ATF4 and ATF5) (Shpilka and Haynes, 2018; Kang et al., 2022). In response to ER stress, protein kinase RNA (PKR)-like ER kinase (PERK), a transmembrane ER protein, is activated and phosphorylates the eukaryotic translation initiator factor 2α (eIF2α) (Shpilka and Haynes, 2018). The phosphorylation of eIF2α will limit protein translation to reduce protein load in the ER; however, this leads to the selective expression of the three bZIP transcription factors (CHOP, ATF4, and ATF5) and further downstream signaling for the UPR^mt^ (Shpilka and Haynes, 2018). The activation of PERK/eIF2α has also been demonstrated to lower ROS production from the mitochondrial electron transport chain. While the functions of protein players in UPR^mt^ and their signaling are still yet to be fully elucidated, we can look at some of the signaling cascades of the UPR^ER^ through ER players, such as PERK and ATF4.

The high metabolic need for RGCs means that any mitochondrial dysfunction can be highly detrimental to these neurons due to a compromise in ATP production and oxidative damage. In glaucoma, changes to mitochondrial dynamics, bioenergetics, metabolism, and structure have been observed (Ju et al., 2022). Dynamin-related GTPases, optic atrophy type 1 (OPA1) and dynamin-related protein 1 (DRP1), regulate mitochondrial dynamics: fusion and fission, with DRP1 being the main effector involved in mitochondrial fission. It has been shown that the overproduction of ROS and increased Ca^2+^ signaling can cause the oxidation of cysteine residues on DRP1, which promotes DRP1 assembly into the ring-like oligomers, initiating more mitochondrial fission, leading to further ROS accumulation in the mitochondria (NavaneethaKrishnan et al., 2020; Yang S. et al., 2020).

Both oxidative stress and reduced mitochondrial respiration have been observed in patients with POAG (Abu-Amero et al., 2006; Ju et al., 2022). In the DBA/2J mouse model of inherited glaucoma, RGCs showed abnormal changes in mitochondrial structure and increased mitochondrial number, demonstrating decreased energy production from these damaged mitochondria and favorability towards fission (Coughlin et al., 2015; Kim et al., 2015; Ju et al., 2022). These abnormal changes in the mitochondria can lead to a type of autophagy called mitophagy to remove the damaged mitochondria. Impaired mitophagy has been observed in both rat and mouse models of glaucoma with decreased levels of the lysosome-associated membrane protein 1 (LAMP1) and increased mitophagosome formation, indicating that the mitochondria are not being recycled efficiently by the lysosomes (Coughlin et al., 2015; Dai et al., 2018; Ju et al., 2022).

### 2.2. ER stress

In addition to functioning as the major Ca^2+^ store and acting in the UPR pathway, the ER also participates in lipid and steroid synthesis and drug metabolism, among many other functions (Schwarz and Blower, 2016; Hurley et al., 2022). Typical functions of the ER can also be disrupted by nutrient deprivation, hypoxia, and even oxidative stress (Lin et al., 2008; Hurley et al., 2022). ER stress and UPR activation have been implicated in RGC degeneration in pre-clinical models of glaucoma (Doh et al., 2010; Hu, 2016). When these dysfunctions occur, ER stress further triggers the UPR^ER^ and two other signaling pathways: ER overload response (EOR) and ER-associated degradation (ERAD) (Hurley et al., 2022).

The ER unfolded protein response pathway decreases the amount of unfolded proteins through the expansion of the ER membrane and the reduction of protein entries into the ER, along with other mechanisms (Hetz, 2012). Three major transmembrane ER proteins that act as stress sensors are involved in the UPR^ER^: inositol-requiring protein 1α (IRE1α), activating transcription factor 6 (ATF6), and PERK (Ron and Walter, 2007; Hetz, 2012). To reduce the influx of proteins into the ER, PERK will also inhibit general protein translation through the phosphorylation of eIF2α, like the mechanism of the UPR^mt^. At the same time, IRE1α will degrade mRNA transcripts that code for certain ER-located proteins through regulated IRE1-dependent decay (RIDD) (Han et al., 2009; Hetz, 2012; Kang et al., 2022). Activation of IRE1α will also lead to further downstream expression of spliced transcription factor X box-binding protein 1 (XBP1s), with its gene products participating in the ERAD response (Lee et al., 2003; Acosta-Alvear et al., 2007; Hetz, 2012).

Under ER stress, ATF6 activation leads to the regulation of genes encoding chaperone proteins, ERAD components, and XBP1 (Lee et al., 2002; Yamamoto et al., 2007; Hetz, 2012). ATF6 is exported to the Golgi apparatus, where it will be mostly digested by proteases to release a fragment that will be translocated to the nucleus to initiate the transcription of proteins involved in abnormal protein cleanup. Unlike PERK and IRE1α, ATF6 does not stop the influx of proteins into the ER but increases the ER’s protein processing and degradation capacity (Kang et al., 2022).

Since glaucoma is a chronic condition, this can also put the RGCs under long-term ER stress. If the three signal transduction pathways (UPR^ER^, EOR, ERAD) cannot restore equilibrium in the ER, they will induce apoptosis, which has been proposed to happen in a caspase-dependent manner (Hetz, 2012).

### 2.3. Inflammation

Another factor implicated in glaucomatous RGC degeneration is the presence of neuroinflammation. Pro-inflammatory cytokines, such as tumor necrosis factor α (TNF-α), have been detected in mechanically strained RGCs, the optic nerve crush model, and glaucomatous human eyes (Morzaev et al., 2015; Lim et al., 2016; Jassim et al., 2021). In glaucomatous inflammatory signaling, the NOD-, LRR-and pyrin domain-containing protein 3 (NLRP3) inflammasome is a significant player (Yerramothu et al., 2018; Jassim et al., 2021). Common triggers for this protein complex can include extracellular ATP and ROS from cell damage and damaged mitochondria (Zhou et al., 2011; Gombault et al., 2012; Yin et al., 2016; Jassim et al., 2021). IOP elevation has been shown to activate the NLRP3 inflammasome leading to the loss of RGCs (Pronin et al., 2019; Jassim et al., 2021). Given that many of these cellular stress responses from both the mitochondria and ER are interlinked due to common stressors such as excessive ROS, ischemia, and ocular hypertension, there must be some communication exchanged between the two organelles.

## 3. Structure and function of mitochondria-associated ER membranes (MAMs)

**Figure 1:**
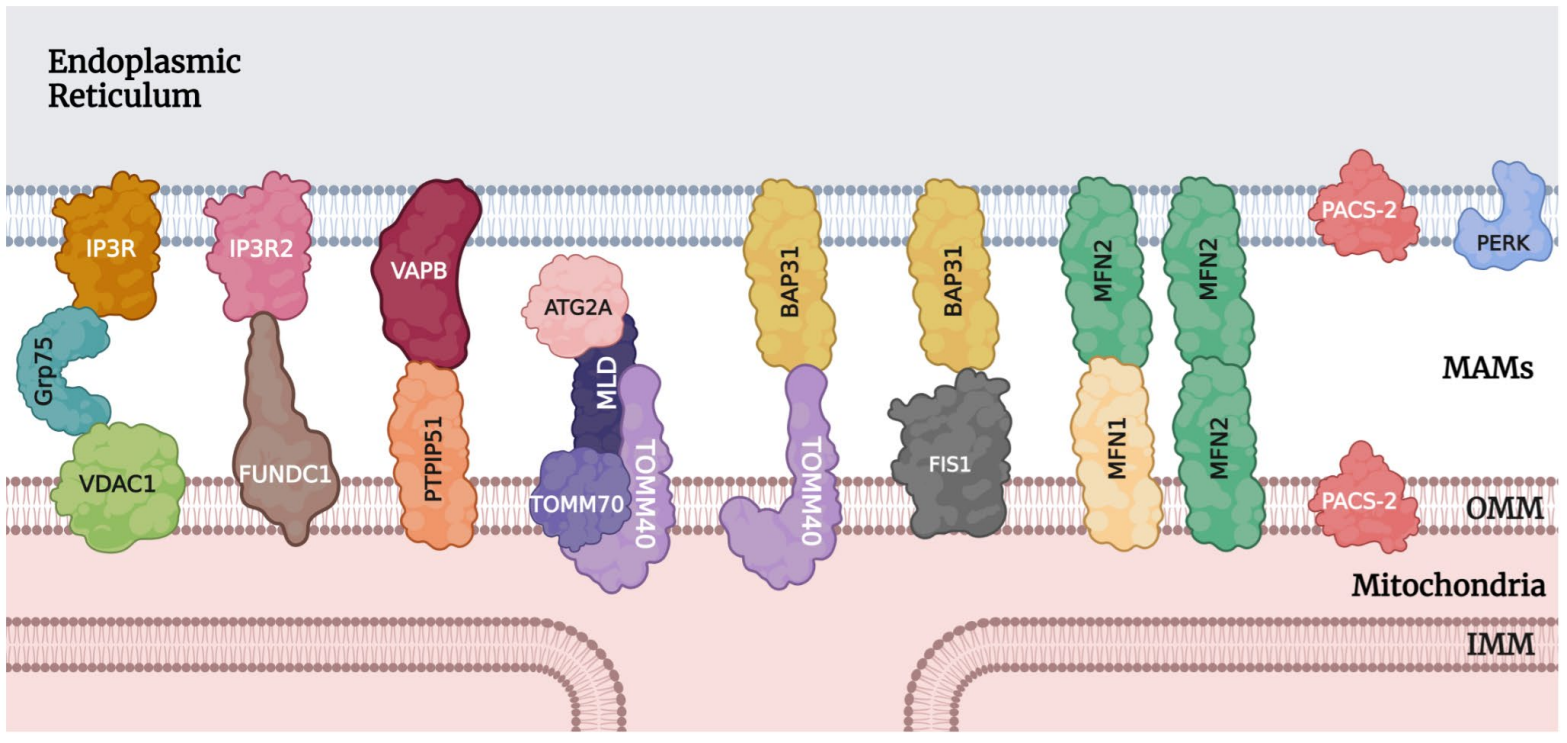
Tethering proteins and other notable protein players at MAMs. IP3R/IP3R2, inositol 1,4,5-triphosphate receptor; Grp75, glucose-regulated protein 75; VDAC1, voltage-dependent anion-selective channel 1; FUNDC1, FUN14 domain containing 1; VAPB, vesicle-associated membrane protein-associated protein B/C; PTPIP51, protein tyrosine phosphatase-interacting protein 51; ATG2A, autophagy-related 2A; MLD, MAMs localization domain; TOMM40/70, translocase of outer mitochondrial membrane 40 and 70; BAP31, B cell receptor-associated protein 31; FIS1, mitochondrial fission 1; MFN1/2, mitofusin 1 and 2; PACS-2, phosphofurin acidic cluster sorting protein 2; PERK, protein kinase RNA (PKR)-like ER kinase; OMM, outer mitochondrial membrane; IMM, inner mitochondrial membrane.

MAMs act as a communication hub by mediating the transport of signaling molecules between the mitochondria and ER, regulating different signaling pathways to ensure functional crosstalk between the two. An interaction between mitochondria and the ER was first observed in rat liver cells by Bernhard et al. (1952) and Yang M. et al., (2020). In 1990, Vance coined the term “mitochondria-associated membranes (MAMs)” (Vance, 1990; Yang M. et al., 2020). Mass spectrometry analysis in 2016 revealed more than 1,000 proteins in the MAMs fragments (Sala-Vila et al., 2016; Yang M. et al., 2020). In 2017, 68 proteins were found to be localized to the MAMs (Hung et al., 2017; Yang M. et al., 2020). Proteins at this location are grouped according to their functions, such as inositol 1,4,5-triphosphate receptor (IP3R) and voltage-dependent anion-selective channel 1 (VDAC1) for Ca^2+^ transport (Tubbs et al., 2014; D’Eletto et al., 2018) and autophagy-related 2/5/14 (ATG2/5/14) for autophagosomes formation (Figure 1; Hamasaki et al., 2013; Yang M. et al., 2020).

There are various MAMs protein tethers in mammalian cells. Some of the ones significant to the context of MAMs in glaucoma will be discussed here. Yang M. et al. (2020) identified the most important protein complex involved in ER-mitochondria coupling as IP3R/Grp75/VDAC1. Inositol 1,4,5-triphosphate receptors (IP3Rs) are essential calcium channels that can significantly modulate cellular metabolism and autophagy (Kania et al., 2017; Valladares et al., 2018; Yang M. et al., 2020). On the outer mitochondrial membrane (OMM), VDAC1 mediates the uptake of Ca^2+^ into the mitochondria (Lipper et al., 2019; Yang M. et al., 2020). Grp75 is a member of the heat shock protein 70 family. It binds to IP3R and VDAC1 to stabilize the protein complex and improve Ca^2+^ transport (Xu et al., 2018; Yang M. et al., 2020). Another player in the Ca^2+^ transport process is the sigma-1 receptor which modulates IP3R to increase ATP production (Hayashi and Su, 2007; Tagashira et al., 2014; Yang M. et al., 2020). Also acting as a marker of MAMs, the IP3R-VDAC1 protein complex is the core structure for Ca^2+^ transport. Two other proteins that function in calcium transport and protein signaling are calnexin and PERK through modulations of ER calcium channels (Yang M. et al., 2020; Bhardwaj et al., 2022). After entering the mitochondria through VDAC on the OMM, calcium ions can bind to the regulatory subunit mitochondrial calcium uptake 1 (MICU1), causing the OPA1 cap over the cristae junction opening on the inner mitochondrial membrane (IMM) to unblock transiently. This temporary opening allows for a rapid influx of calcium ions into the matrix through the mitochondrial calcium uniporter (MCU) complex (MCUC) on the cristae membranes, which is composed of pore-forming subunit MCU, regulatory subunits MICU1, MICU2, essential MCU regulator (EMRE), and associated proteins. The MCUC plays a vital role in many cellular processes, including energy production, calcium signaling, and cell death (Gottschalk et al., 2022).

For unfolded protein response and vesicle trafficking, vesicle-associated membrane protein-associated protein B/C (VAPB) on the ER membrane plays an important role (Lee and Min, 2018; Yang M. et al., 2020). VAPB can form a complex with protein tyrosine phosphatase-interacting protein 51 (PTPIP51) and mediate calcium ion transport and autophagy at the MAMs (De Vos et al., 2012; Gomez-Suaga et al., 2017; Yang M. et al., 2020). Other players involved in autophagosome formation also include autophagy-related 2A (ATG2A), MAMs localization domain (MLD), and the translocase of outer mitochondrial membrane 40 and 70 (TOMM40/70) (Yang M. et al., 2020). Another group of protein complexes of importance is BAP-31 with TOMM40 or FIS1. B cell receptor-associated protein 31 (BAP31) is an ER transmembrane protein that participates in apoptosis through calcium signaling and the ERAD pathway (Niu et al., 2017; Yang M. et al., 2020). On the mitochondrial side, TOMM40 promotes the translocation of proteins into the mitochondria (Gonzalez Montoro et al., 2018; Yang M. et al., 2020), while mitochondrial fission 1 (FIS1) interacts with BAP31 to activate its cleavage into the pro-apoptotic p20BAP31 (Iwasawa et al., 2011; Namusamba et al., 2021).

Outside of protein complexes, there are also individual proteins that connect the ER and mitochondria. The Mmm1 protein, for example, is essential for stabilizing the MAMs and influencing calcium ion homeostasis in neurons (Hirabayashi et al., 2017; Yang M. et al., 2020). Another protein, phosphofurin acidic cluster sorting 2 protein (PACS-2), is also involved in MAMs stability, apoptosis, and autophagy (Herrera-Cruz and Simmen, 2017; Moulis et al., 2019; Li et al., 2020; Yang M. et al., 2020). Loss of PACS-2 can lead to the destruction of MAMs and dysregulation of mitophagy (Moulis et al., 2019; Yang M. et al., 2020). Parkin, a protein involved in mitophagy signaling, has been shown to be involved in maintaining MAMs integrity by affecting the ubiquitination of mitofusin 2 (MFN2), a protein located in the MAMs that participates in mitochondrial fusion.

## 4. Role of MAMs in retinal ganglion cell degeneration

It has been shown in several studies that there is a crosstalk between the UPR^mt^ and UPR^ER^ (Lu et al., 2014; Rainbolt et al., 2014). One way this has been demonstrated is through the PERK signaling pathway that plays a role in ROS-induced apoptosis (Verfaillie et al., 2012, 2013; Kang et al., 2022). The knockout of PERK in murine embryonic fibroblasts was found to result in a disturbance to the ER-mitochondria association and decreased ROS signaling and Ca^2+^ influx from the ER to the mitochondria (Liu et al., 2013; Kang et al., 2022). In a rat model of glaucoma (induction of ocular hypertension), there was a significant increase in the expression of Grp78 and CHOP-two proteins in the PERK signaling pathway (Doh et al., 2010; Hurley et al., 2022). Inhibition of the PERK-eIF2-CHOP pathway also demonstrated RGC soma and axon protection in various mouse models of glaucoma (Bhattarai et al., 2021; Hurley et al., 2022). Through different mouse models of optic neuropathies (traumatic optic nerve injury and glaucoma), Yang et al. (2016) showed that manipulation of the UPR^ER^ pathway by inhibiting eIF2-CHOP and activating XBP1 also promoted RGC soma and axons survival and even preserved visual function (Yang et al., 2016; Hurley et al., 2022).

As discussed earlier, a significant function of MAMs is regulating Ca^2+^ signaling. Ca^2+^ signaling is crucial for cell survival. When a cell is stimulated under normal conditions, the ER releases calcium ions through IP3Rs and ryanodine receptors (RyRs) to be taken up by the mitochondrial matrix to activate the tricarboxylic acid cycle for ATP production (Duchen, 2000). However, elevated calcium levels have been shown in RGC apoptosis induced by hydrostatic pressure (Sappington et al., 2009; Hurley et al., 2022). In ER stress, calcium is also released through IP3Rs and RyRs (Deniaud et al., 2008; Hurley et al., 2022). The intake of calcium and accumulation in the mitochondrial matrix leads to mitochondrial permeability transition pore opening, ROS production, and disruption of ATP production, starting a vicious cycle of damage to the mitochondria (Peng and Jou, 2010; Hurley et al., 2022). Increased ROS can also stimulate an increase in the intracellular Ca^2+^ concentration and activate the UPR (Gorlach et al., 2015; Hurley et al., 2022). Like UPR^ER^, the accumulation of misfolded proteins in the ER will also trigger EOR (Hurley et al., 2022). When EOR is activated, calcium is released from the ER and initiates ROS production. This will lead to the activation of nuclear factor kappa-light-chain-enhancer of activated B-cells (NF-κB), which can initiate both the canonical and alternative inflammatory response pathways (Liu et al., 2017; Bhattarai et al., 2021; Hurley et al., 2022).

One of the most important and most studied mitophagy pathways is the PTEN-induced putative kinase (PINK1) and parkin pathway. In pathological conditions, PINK1 accumulates on the OMM and causes the phosphorylation of ubiquitin, leading to parkin recruitment. Activated parkin can polyubiquitinate VDAC1 and other proteins that bind to LC3 to initiate autophagosome formation and mitophagy (Tanida et al., 2008; Schaaf et al., 2016; Wang et al., 2020). Through VDAC1’s involvement in Ca^2+^ signaling, parkin will also promote Ca^2+^ transport into the mitochondria and increase ATP production (Duchen, 2000; Brookes et al., 2004; Cali et al., 2013; Yang M. et al., 2020).

FUNDC1-mediated mitophagy is another mitophagy pathway involving MAMs. This pathway relies on the FUN14 domain containing 1 (FUNDC1), a MAM-localized protein, which interacts with IP3R2 and facilitates IP3R-dependent Ca^2+^ release from the ER to the mitochondria and cytosol (Wu et al., 2017; Yang M. et al., 2020). When the expression of FUNDC1 decreases, Ca^2+^ levels reduce in both mitochondria and cytosol, leading to mitochondrial dysfunction through Ca^2+^-sensitive cAMP-response element binding protein (CREB) and disrupting MAMs protein tethers (Wu et al., 2017; Yang M. et al., 2020). Under normal conditions, FUNDC1 can interact with OPA1 for the purpose of mitochondrial fusion (Chen et al., 2016; Yang S. et al., 2020). The loss or alteration of OPA1 expression has been shown to result in the disturbance of calcium homeostasis, depletion of cristae junction, and increased mitochondrial fission, such as seen in IOP elevated DBA/2J mice (Ju et al., 2008; Kushnareva et al., 2013). In response to hypoxic conditions due to insufficient oxygen transport, glaucoma-related axonal transport deficits occur, and FUNDC1 increases significantly in MAMs and recruits DRP1 to promote mitochondrial fission, which can initiate mitophagy (Wu et al., 2016; Yang M. et al., 2020). The initiation of mitophagy will then cause the recruitment of another player: the NLRP3 inflammasome. While normally present in the ER, when NLRP3 is activated in response to mitophagy/autophagy or ROS, it relocates from the ER to MAMs and connects to the adaptor protein ASC to initiate the assembly of the NRLP3 inflammasome (Green et al., 2011; Zhou et al., 2011; Jassim et al., 2021). While the involvement of MAMs in glaucomatous inflammation has not been fully understood, MAMs play a role in initiating inflammation as part of the cellular defense mechanism (Missiroli et al., 2018).

In conclusion, the evaluation of mitochondrial dysfunction and ER stress has been studied extensively in glaucoma research in various tissues, including the trabecular meshwork at the front of the eye to the retina and the optic nerve head at the back of the eye. Some of the multiple pathways involved in these molecular pathologies have also been studied, including PERK signaling, which modulates functions for both organelles. Other pathways include PINK1/parkin, CREB, and apoptotic signaling pathways. However, the study of MAMs in the context of glaucoma has yet to be done as extensively. Studies have shown the involvement of MAMs in these signaling pathways, some of which are essential to their functions. Since the collaborative activities between the mitochondria and ER have been demonstrated, future studies in RGC degeneration in the context of glaucoma should evaluate MAMs markers in addition to markers of the mitochondria and ER.

## Author contributions

JP: writing—original draft preparation. JP and DS: writing—review and editing. All authors contributed to the article and approved the submitted version.

## Funding

This research was funded by National Eye Institute (EY029823).

## Conflict of interest

The authors declare that the research was conducted in the absence of any commercial or financial relationships that could be construed as a potential conflict of interest.

## Publisher’s note

All claims expressed in this article are solely those of the authors and do not necessarily represent those of their affiliated organizations, or those of the publisher, the editors and the reviewers. Any product that may be evaluated in this article, or claim that may be made by its manufacturer, is not guaranteed or endorsed by the publisher.

## Figures and Tables

**Table 1:** 

ATFS-1	Activating transcription factor associated with stress
ATF4/5/6	Activating transcription factor 4/5/6
ATG2/2A/5/14	Autophagy-related 2/2A/5/14
bZIP	Basic leucine zipper
BAP31	B cell receptor-associated protein 31
CREB	Ca^2+^-sensitive cAMP-response element binding protein
CHOP, also known as DDIT3	C/EBP homologous protein
DRP1	Dynamin-related protein 1
ER	Endoplasmic reticulum
ERAD	ER-associated degradation
EOR	ER overload response
EMRE	Essential MCU regulator
eIF2α	Eukaryotic translation initiator factor 2α
FUNDC1	FUN14 domain containing 1
IMM	Inner mitochondrial membrane
IP3R	Inositol 1,4,5-triphosphate receptor
IRE1α	Inositol-requiring protein 1α
IOP	Intraocular pressure
RIDD	IRE1-dependent decay
Grp75/78	Glucose-regulated protein 75/78
LAMP1	Lysosome-associated membrane protein 1
MLD	MAMs localization domain
MAMs	Mitochondria-associated ER membranes
MCUC	Mitochondrial calcium uniporter (MCU) complex
MICU1/2	Mitochondrial calcium uptake 1/2
FIS1	Mitochondrial fission 1
MFNN1/2	Mitofusin 1 and 2
NLRP3	NOD-, LRR-and pyrin domain-containing protein 3
NF-κB	Nuclear factor kappa-light-chain-enhancer of activated B-cells
OPA1	Optic atrophy type 1
OMM	Outer mitochondrial membrane
PACS-2	Phosphofurin acidic cluster sorting protein 2
POAG	Primary open-angle glaucoma
PERK	Protein kinase RNA (PKR)-like ER kinase
PTPIP51	Protein tyrosine phosphatase-interacting protein 51
PINK1	PTEN-induced putative kinase
ROS	Reactive oxygen species
RGCs	Retinal ganglion cells
RyRs	Ryanodine receptors
TOMM40/70	Translocase of outer mitochondrial membrane 40/70
TNF-α	Tumor necrosis factor α
UPR^ER/mt^	Unfolded protein response (ER/mitochondrial)
VAPB	Vesicle-associated membrane protein-associated protein B/C
VDAC1	Voltage-dependent anion-selective channel 1
XBP1	X box-binding protein 1
